# Ischemic stroke – the challenge continues

**DOI:** 10.3325/cmj.2016.57.217

**Published:** 2016-06

**Authors:** Vida Demarin, Hrvoje Budinčević

**Affiliations:** 1 International Institute for Brain Health, Zagreb, Croatia *vida.demarin@gmail.com*; 2 Stroke and Intensive Care Unit, Department of Neurology, Sveti Duh University Hospital, Zagreb, Croatia; 3 School of Medicine, J.J. Strossmayer University of Osijek, Osijek, Croatia

Under the leadership of the World Health Organization, many countries have recently agreed to start reducing the burden of avoidable non-communicable diseases (NCD) (1). Most NCD deaths are the result of cardiovascular diseases and they mostly occur in low and middle income countries (2)

Furthermore, epidemiological data regarding stroke burden in Central and Eastern European countries are not acceptable – the incidence and prevalence of stroke in Central and Eastern European countries are still higher than in Western European countries (3-5). Despite improvement in technical support, some countries in the region are struggling with basic organization problems such as forming stroke units (3), which have been clearly shown to save lives in both ischemic and hemorrhagic stroke (6,7). According to the European Stroke Organization (ESO) guidelines, we may divide stroke units in primary stroke units and comprehensive stroke units (8). In parallel with the certification of stroke units by ESO or other authorities, countries should encourage the founding of stroke units based on the principles. But first of all, it is necessary to have dedicated and trained stroke physicians.

Before the introduction of intravenous thrombolysis, we witnessed treatment nihilism in acute stroke treatment (9). Nowadays, the management of acute stroke has evolved and includes intravenous thrombolysis, mechanical thrombectomy, and decompressive craniotomy (10). Unfortunately, intravenous thrombolysis for ischemic stroke is still underutilized due to various exclusion criteria, with early and narrow time-window and possible hemorrhagic complications (11). Recently, the usage of low dose alteplase in stroke patients has been confirmed as safe and feasible (12). This finding might increase the number of thrombolysis cases in low and middle income countries because of financial health care system limitations. Mechanical thrombectomy trials now show clear and significant outcome results in selected patients with proximal cerebral artery occlusion (13). According to decompresive craniectomy trials and current guidelines, these treatment options are reserved for the patients with malignant middle cerebral artery infarction and for selected patients with cerebelar infarctions (14).

Unfortunately, these treatments are not available in many stroke centers due to organizational, financial, and technical constraints (3). As it was mentioned before, in many countries we still do not have full coverage by stroke units, and the treatments with intravenous thrombolysis are not a routine procedure in European countries (especially eastern European countries) (3).

Evident improvement is present in the field of stroke prevention. Pharmacological treatments are widely available in many industrialized countries, as opposed to developing countries (15). The recent introduction of direct oral anticoagulants has been based on a clinical trial that showed superiority, or at least non-inferiority, to warfarin, which was the golden standard for stroke prevention in patients with atrial fibrillation (16). Novel studies also suggest that the most common cause of cryptoembolic stroke or embolic stroke of undetermined source is paroxysmal atrial fibrillation, which can be detected by prolonged ECG monitoring (17). Non-pharmacological treatments such are healthy lifestyle with appropriate diet (Mediterranean diet) and everyday physical activity are highly recommended (10,18).

Despite all these improvements, stroke is still the leading cause of disability and the third cause of death in the world (19). It is indeed difficult to counteract the fact that two millions of neurons die every minute during anterior circulation stroke (20). Since neuroprotection agents and procedures still have not showed adequate benefit, further translational research is necessary to improve stroke treatments and save brain from irreversible damage (21).

In the following years, we should be **faster** in stroke treatment, with **higher** numbers of stroke units and **stronger** links among stroke centers to achieve our goals regarding acute stroke treatment. In stroke prevention, non-pharmacological and pharmacological treatment should be used synergistically.
